# HDL levels modulate the impact of type 2 diabetes susceptibility alleles in older adults

**DOI:** 10.1186/s12944-024-02039-7

**Published:** 2024-02-22

**Authors:** Siobhán O.’ Sullivan, Cynthia Al Hageh, Andreas Henschel, Stephanie Chacar, Antoine Abchee, Pierre Zalloua, Moni Nader

**Affiliations:** 1 Department of Biological Sciences, College of Medicine and Health Sciences, Khalifa University, Abu Dhabi, United Arab Emirates; 2 Department of Computer Science, College of Engineering, Khalifa University, Abu Dhabi, United Arab Emirates; 3 Department of Medical Sciences, College of Medicine and Health Sciences, Khalifa University, PO Box 127788, Abu Dhabi, United Arab Emirates; 4 Faculty of Medicine, University of Balamand, Balamand, Lebanon; 5 Harvard T.H. Chan School of Public Health, Boston, MA USA; 6 Department of Public Health and Epidemiology, College of Medicine and Health Sciences, Khalifa University, Abu Dhabi, United Arab Emirates

**Keywords:** Older age groups, Genetic variants, HDL, Diabetes risk

## Abstract

**Background:**

Type 2 Diabetes (T2D) is influenced by genetic, environmental, and ageing factors. Ageing pathways exacerbate metabolic diseases. This study aimed to examine both clinical and genetic factors of T2D in older adults.

**Methods:**

A total of 2,909 genotyped patients were enrolled in this study. Genome Wide Association Study was conducted, comparing T2D patients to non-diabetic older adults aged ≥ 60, ≥ 65, or ≥ 70 years, respectively. Binomial logistic regressions were applied to examine the association between T2D and various risk factors. Stepwise logistic regression was conducted to explore the impact of low HDL (HDL < 40 mg/dl) on the relationship between the genetic variants and T2D. A further validation step using data from the UK Biobank with 53,779 subjects was performed.

**Results:**

The association of T2D with both low HDL and family history of T2D increased with the age of control groups. T2D susceptibility variants (rs7756992, rs4712523 and rs10946403) were associated with T2D, more significantly with increased age of the control group. These variants had stronger effects on T2D risk when combined with low HDL cholesterol levels, especially in older control groups.

**Conclusions:**

The findings highlight a critical role of age, genetic predisposition, and HDL levels in T2D risk. The findings suggest that individuals over 70 years who have high HDL levels without the T2D susceptibility alleles may be at the lowest risk of developing T2D. These insights can inform tailored preventive strategies for older adults, enhancing personalized T2D risk assessments and interventions.

**Supplementary Information:**

The online version contains supplementary material available at 10.1186/s12944-024-02039-7.

## Background

Heritability of Type 2 Diabetes (T2D) ranges from 30–70% and this variability maybe due to population differences as well as the epidemiological approaches employed. Family history doubles the risk for siblings and triples it for first degree relatives [1, 2].

Genetic studies, particularly GWAS (Genome Wide Association Study) have deepened our understanding of T2D’s genetic susceptibility across various populations. In the Lebanese population, *TCFL2* (Transcription factor 7-like 2) and *CDKAL1* (Cdk5 regulatory associated protein 1-like 1) genes have previously been reported as having a role in T2D disease susceptibility [3]. Variants within the *CDKAL1* locus significantly increase T2D risk as shown in several replication studies in diverse populations [4]. Several GWAS studies report the association of several genes related to lipid metabolism [5–7]. Increased expression of *GRINA *(Glutamate Receptor, Ionotropic, N-Methyl D-Aspartate-Associated Protein 1) and *GPR146* (G Protein-Coupled Receptor 146) are associated with reduced levels of LDL (Low Density Lipoprotein)-cholesterol in plasma and an increase in plasma total cholesterol levels in humans, respectively [8]. In *GPR146* deficient mice, there is a significant decrease in the levels of total- VLDL (Very Low Density Lipoprotein), - HDL (High Density Lipoprotein), and - LDL -cholesterol [9].

The exact protective role of HDL against T2D remains debated. Abnormalities in HDL structure impair its function resulting in alterations in glucose and lipid metabolism, protein glycation, lipid oxidation, and inflammation. The diverse biological activities of HDL particles are closely associated with their varied functions in vivo, and these are pertinent to the pathophysiology of hyperglycaemia, inflammation, dyslipidemia, and vascular dysfunction in diabetes [10, 11]. They contribute to the maintenance and repair of endothelial tissues and regulate aspects of the innate and adaptive immune system responses. The wide range of functions are frequently mediated through signal transduction pathways and various cell types e.g. pancreatic cells, adipocytes, smooth muscle cells, and immunoinflammatory cells [12]. Disruption of signal pathways can lead to the inhibition of the c-Jun N-terminal kinase pathway which has downstream effects on glycaemic control as well as insulin synthesis and secretion from pancreatic beta cells. HDLs either interact with many cell receptors activating different pathways or they are internalized. HDL components which include ApoA1 and S1P activate the Akt/eNOS pathway through binding to receptors that inhibit apoptosis in endothelial cells hereby maintaining their integrity [13–17].

Many age-related diseases have features that resemble premature ageing [18]. Indeed several chronic diseases and ageing share pathophysiological processes underpinned by disruption of signal pathways at the molecular and cellular level [19–21]. Ageing increases T2D risk, primarily due to the impact of agieng on insulin secretion impairment and increased insulin resistance, often associated with obesity and sarcopenia [22]. Adipose tissue characteristically has increased cell senescence in obesity, T2D, and in the earliest stages of ageing [23, 24]. T2D incidence increases with age, occurring most commonly between 55–59 years and manifesting slightly earlier in men than in women [25, 26]. The US sees 9.9 cases among every 1000 individuals annually for the age group 45–64, dropping to 8.8 for those aged over 65. Those with exceptional longevity and their descendants showcase better measures of physical function, lower cardiovascular risk factors, higher HDL levels, and lower triglycerides levels [27, 28]. This aligns with earlier findings indicating that the children of individuals with extended lifespans exhibit better lipid profiles compared to control groups [28, 29]. T2D results from genetic and environmental interactions. It is probable that specific environmental factors amplify the effects of genetic factors that contribute to individual susceptibility. Similarly, with identical lifestyles, genetic differences can make some more prone to developing T2D. Ageing pathways and cellular senescence aggravate metabolic diseases including T2D. HDL is suggested to serve a protective role against T2D in ageing populations, offsetting certain genetic susceptibilities.

While there is considerable evidence supporting the protective role of HDL against development of T2D, the exact mechanism remains unclear. What is known that T2D causes changes to the HDL proteome and lipidome, which affect a number of processes apart from altered glucose homeostasis. Studies on adipose tissue endocrine function and adiponectin, a marker of adipose health and its role in metabolic processes (including HDL biogenesis) that underpin T2D is promising [27, 30].

Limited research has been done on the protective role of HDL in older individuals including the molecular pathways and mechanisms that underlie reduced risk of T2D and favorable glucose management. This study examined the relationship between T2D susceptibility variants, HDL cholesterol levels, and T2D risk in older Lebanese adults, aiming to identify those with lower T2D risk. Focusing on the interaction between HDL, genetic risk factors, and ageing, the study provides an insight which may be transferable to other age-related metabolic diseases expanding its novelty beyond T2D research and potentially informing personalized and age-specific prevention or treatment strategies.

## Methods

### Subjects and data collection

This study involved 2,769 patients with type 2 diabetes (T2D), and 3,167 subjects without diabetes aged ≥ 60 years, among whom 2,323 aged ≥ 65 years and 1,545 aged ≥ 70 years (1,527, 1,382, 1,027 and 683 genotyped, respectively) (Supplementary Fig. 1). The gender distribution was as follows: for T2D, 39.2% were female and 60.8% were male; among those without T2D aged ≥ 60 years, 38.6% were female and 61.4% were male; in the ≥ 65 years group without T2D, 39.9% were female and 60.1% were male; and among those aged ≥ 70 years without T2D, 40.7% were female and 59.3% were male. The subjects are within the FGENTCARD, which is curruntly within the CARDIoGRAMplusC4D consortium [3, 31, 32]. The Lebanese American University Institutional Review Board’s ethics committee approved the study. All the participants signed their informed consent before beginning the study, which followed the Helsinki Declaration of 1975. A questionnaire was used to gather epidemiological information including family history (Fx) of diseases (Family history was considered only in patients with first or second degree relatives). Additional clinical information (such as cardiovascular disease, hypertension and hyperlipidemia status), was obtained from patients’ medical charts. Anthropometric measurements such as height, weight, and waist circumference were taken during the patients’ visits. Blood samples were taken from fasting subjects for metabolic profiles and DNA extraction. The COBAS INTEGRA 400 Plus was used to measure levels of HDL-, LDL- cholesterol and triglycerides. The Abbot Architect c1000 was used to measure blood sugar levels.

### Genotyping and statistical analysis

DNA was extracted using previously established methods [3]. Genotyping of 913,353 SNPs was performed on Illumina Human 610 and 660W Quad BeadChips. Previous analyses using the same population showed that population stratification was not a confounder [32, 33]. Additionally, we calculated the genomic inflation factor (λ) from both the Q-Q plot and association analysis, consistently yielding values around 1.077. These results, provide further assurance regarding the absence of population stratification. Subsequent quality control measures were implemented using PLINK 1.9 [34, 35] (www.cog-genomics.org/plink/1.9/), excluding SNPs (Single nucleotide polymorphisms) with ≥ 10% missing genotyping rates, gender discrepancy, < 1% minor allele frequency (MAF) and those deviating from Hardy–Weinberg Equilibrium (*P* < 0.001). These rigorous measures were undertaken to ensure data integrity and minimize potential biases within the dataset. Finally, 244,609 SNPs were analyzed for GWAS.

Statistical analysis used the R package (v4.2.2). The continuous variables were compared with One-Way ANOVA, including age, weight, body mass index (BMI), LDL-cholesterol, HDL-cholesterol, and total-cholesterol, triglyceride levels, glucose, and C-reactive protein (CRP). The chi-squared (χ2) test was used to compare categorical variables. A binomial logistic regression was utilized to assess the relationship between T2D and risk factors such as male gender, female gender, high BMI (categorized as BMI levels ≥ 25 kg/m^2^), low HDL (characterized by HDL levels less than 40 mg/dL for all subjects, < 40 for men and < 50 for women [36]), hypertension, hyperlipidemia, Fx hypertension, Fx T2D, and Fx cardiovascular disease, taking non- diabetic subjects aged ≥ 60, ≥ 65, or ≥ 70 as control groups. In addition, the logistic regression analysis was conducted with stratification by gender, which essentially allowed for gender-specific analyses. This approach was chosen to explore potential gender-specific effects on T2D risk factors. Logistic regression, adjusted for gender, was performed in the additive model. Significance thresholds were defined as suggestive (*P* < 1 × 10^–5^) and genome-wide (*P* < 5 × 10^–8^). A quantile–quantile (Q-Q) plot was conducted to examine the distribution of *P*-value. Regional association plots were generated through LocusZoom web tool (http://locuszoom.sph.umich.edu/locuszoom/) using the hg19/1000 genomes Nov 2014 EUR as reference. A stepwise logistic regression assessed the interplay between T2D-associated SNPs and the influence of low HDL by adding it to the model. While stepwise logistic regression is used to assess the impact of low HDL on SNP associations with T2D, it is important to note however that there is a risk of model overfitting and could potentially overlook interactions with unaccounted factors, influencing the interpretation of the findings*.* In order to investigate whether the identified variants were associated to T2D across a larger population with distinct cases and controls, a validation step was performed using data from the UK Biobank (application number 64823) with 53,779 subjects. T2D subjects were selected based on ICD codes E11.1 to E11.9. Controls were carefully selected as healthy individuals, focusing on the British population. In the replication study, the *CDKAL1* genetic variants (rs4712523, rs7756992, and rs10946403) were specifically targeted for their consistent association with T2D across the control groups in this population aged ≥ 60, ≥ 65, and ≥ 70 years.

## Results

### Association of T2D with risk factors in age-stratified control groups

The study included 2,769 T2D patients and 3,167 controls aged ≥ 60 years, among whom 2,323 were ≥ 65 years and 1,545 were ≥ 70 years. Differences emerged between T2D patients and controls in weight, triglyceride and fasting glucose levels (Table 1).

**Table 1:** Comparison of clinical characteristics between T2D and control groups without diabetes stratified by age

	**T2D**	**noT2D ≥ 60 years**	**noT2D ≥ 65 years**	**noT2D ≥ 70 years**	**P1**	**P2**	**P3**
N	2769	3167	2323	1545			
Age	62.61 (10.25)	70.02 (6.97)	72.96 (5.76)	75.98 (4.65)	< 0.01	< 0.01	< 0.01
BMI (Kg/m^2^)	29.92 (5.37)	29.68 (56.75)	29.95 (66.40)	30.43 (81.54)	0.82	0.98	0.75
Weight (Kg)	80.62 (15.43)	76.01 (14.61)	75.03 (14.33)	73.66 (13.43)	< 0.01	< 0.01	< 0.01
Total Cholesterol (mg/dL)	181.16 (46.78)	185.98 (46.18)	183.46 (45.07)	181.94 (45.35)	< 0.01	0.11	0.63
LDL Cholesterol (mg/dL)	106.23 (40.69)	111.98 (38.77)	109.86 (38.01)	108.81 (38.26)	< 0.01	< 0.01	0.07
HDL Cholesterol (mg/dL)	39.50 (11.94)	44.15 (13.90)	44.52 (14.08)	44.69 (14.45)	< 0.01	< 0.01	< 0.01
Triglyceride (mg/dL)	197.92 (131.79)	155.15 (80.71)	150.88 (75.89)	146.91 (72.39)	< 0.01	< 0.01	< 0.01
Glucose (mg/dL)	159.36 (66.96)	106.43 (33.83)	106.19 (27.74)	105.60 (24.42)	< 0.01	< 0.01	< 0.01
CRP (mg/L)	23.75 (43.67)	15.39 (30.52)	16.03 (32.64)	18.26 (36.73)	< 0.01	0.02	0.16

Data are mean values followed by standard deviation. The *p*-value is generated using One-Way ANOVA. *P*-value is significant if *p*.value < 0.05

*T2D* Type 2 Diabetes, *CRP* C-Reactive Protein, *BMI* Body Mass Index, *HDL* high-density lipoprotein, *LDL* low-density lipoprotein, *P1 p*.value between T2D and noT2D ≥ 60 years, *P2 p*.value between T2D and noT2D ≥ 65 years, *P3 p*.value between T2D and noT2D ≥ 70 years

**Fig. 1:**
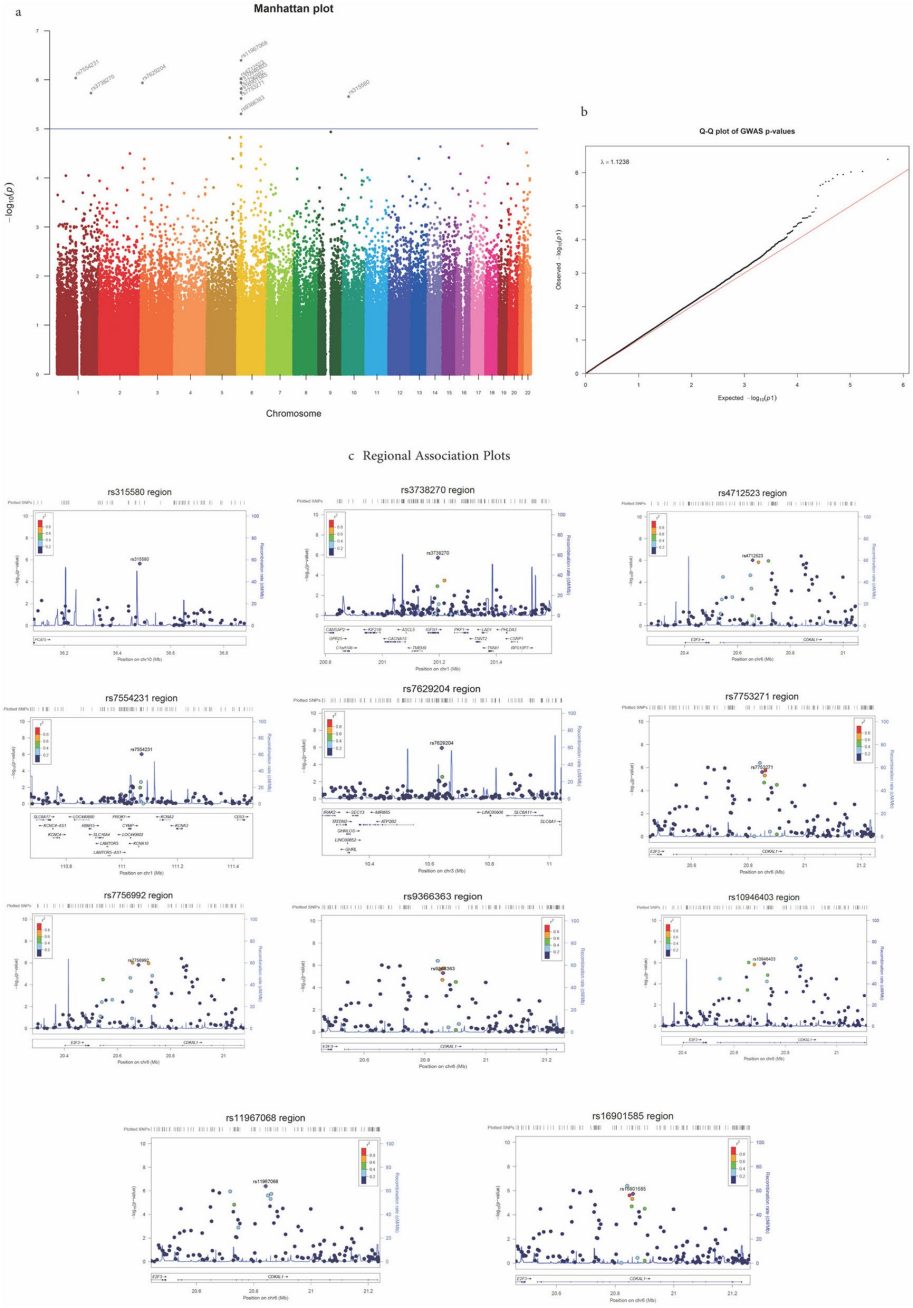
Forest plot of the binomial logistic regression showing evidence of association between T2D and different risk factors stratified by gender, taking (**a**) noT2D ≥ 60 years, (**b**) noT2D ≥ 65 years and (**c**) noT2D ≥ 70 years as control groups. CI: Confidence Interval. T2D: Type 2 Diabetes. Fx: Family history. BMI: Body Mass Index. CVD: Cardiovascular disease

**Fig. 2:**
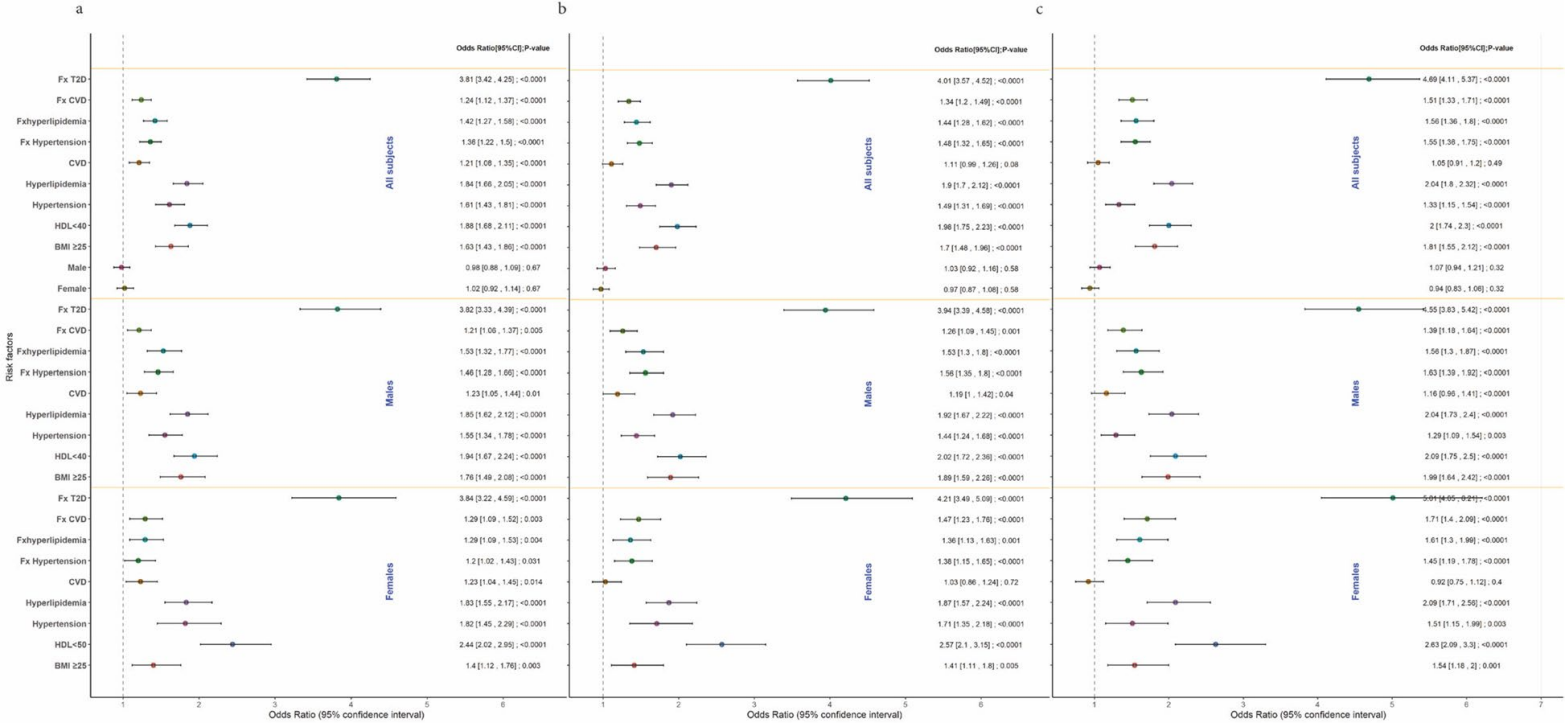
**a** Manhattan plot for the genome-wide association analysis with T2D using individuals aged ≥ 70 years without diabetes as controls. **b** Quantile–Quantile (Q-Q) plot of the GWAS results showing the distribution of *p*-values, plotted against the expected distribution. **c** Regional association plots for the loci associated with T2D among the older adults in the Lebanese population. GWAS: genome-wide association study. T2D: Type 2 Diabetes. **a** X-axis shows chromosomal positions. Y axis represents the − log_10_(*P*-value obtained) by logistic regression analysis (additive model). The horizontal solid blue line indicates the suggestive genome-wide threshold of *P* = 1 × 10^–5^. Each point denotes a Single nucleotide polymorphism (SNP), SNPs with significance *P* < 1 × 10^–8^ are shown above the genome-wide significance in red. **b** This plot shows the association between each tested SNP and the observed -log10 p values, plotted on the vertical axis, compared to the expected –log10 *p* values under the null hypothesis. Each dot on the plot represents a SNP. The genomic control ratio (l) was 1.1238, which indicates that there is no strong effect of systematic error, such as population stratification. **c** Correlations between the SNPs with the lowest *P* value from GWAS (depicted in purple) and nearby SNPs within a 400 kb region. The r^2^ values of the Linkage Disequilibrium (LD) heat map is based on the hg19/1000 genomes Nov 2014 EUR reference set

Logistic regression (Fig. 1) assessed the association between T2D and various risk factors, including gender, high BMI, low HDL, hypertension, hyperlipidemia, Fx hypertension, Fx T2D, and Fx cardiovascular disease. The analysis revealed a positive association between T2D and Fx T2D in all subjects taking as controls individuals without diabetes at all ages (OR = 3.81 (≥ 60), OR = 4.01 (≥ 65) and OR = 4.69 (≥ 70); *P* < 0.001). In females, this association demonstrated an increased risk, with odds ratios of OR = 3.84 (≥ 60), OR = 4.21 (≥ 65) and OR = 5.01 (≥ 70) (*P* < 0.0001). The odds ratios for Fx T2D increased successively with age of control groups. Also, T2D correlated positively with low HDL, particularly in females and with older controls (in females: OR = 2.44 (≥ 60), OR = 2.57 (≥ 65) and OR = 2.63 (≥ 70); *P* < 0.0001). Fx T2D was notably higher in T2D cases (69.4%) compared to control groups (37.7%, 36.1%, and 32.5%, respectively) (Supplementary Table 1, Fig. 2a). T2D cases had lower HDL cholesterol levels than the controls (39.40 mg/dl, 44.15, 44.52 and 44.69 mg/dl, respectively (*P* < 0.01)) (Table 1, Supplementary Fig. 2). This was replicated in the UK Biobank (*P* < 0.01) (Supplementary Fig. 3). Lower HDL levels (< 40mg/dl) were associated with T2D while higher HDL levels (≥ 40 mg/dl) were associated with age-specific control groups (Supplementary Table 1, Supplementary Fig. 2).

### Genetic insights into T2D: GWAS and replication in UK Biobank

GWAS analysis identified SNPs associated with T2D. In ≥ 60 controls, 4 *CDKAL1* loci showed positive associations (rs7756992, rs9366354, rs10946403, rs4712523; OR = 1.39, 1.34, 1.34 and 1.36, respectively (*P* < 1 × 10^–5^ In all)) while 3 other *CDKAL1* loci showed negative associations (rs10946415, rs4710944, rs9465895; OR = 0.71, 0.73 and 0.73, respectively (*P* < 1 × 10^–5^ In all)) with T2D (Supplementary Table 2, Supplementary Fig. 4). In addition, one SNP (*LOC105369844*, rs11180649) was negatively associated with T2D (OR = 0.71, *P* = 6.70 × 10^–6^) and two *TCF7L2* loci were positively associated (rs7903146 and rs7901695; OR = 1.35 and 1.34 respectively (*P* < 1 × 10^–5^)). In ≥ 65 controls, 3 *CDKAL1* loci had more significant associations with T2D (rs7756992, rs4712523, and rs10946403; OR = 1.40, 1.39, 1.38 and *P* = 2.01 × 10^–06^, 3.24 × 10^–06^ and 8.86 × 10^–06^, respectively) (Supplementary Fig. 5). One locus in *CDKAL1* (rs10946415, OR = 0.71; *P* = 4.89 × 10^–06^) and one *IGFN1* locus (rs3738270, OR = 0.74; *P* = 8.16 × 10^–06^) were negatively associated with T2D. In ≥ 70 controls (Fig. 2), seven *CDKAL1* SNPs were associated with T2D, with rs11967068 showing the strongest association (OR = 1.84, P = 4.03 × 10^–07^), and rs20858852 showing negative association with T2D (OR = 0.68, *P* = 4.95 × 10^–6^). SNP *IGFN1* (rs3738270) was negatively associated, and one *ATP2B2* SNP (rs7629204) was positively associated with T2D (OR = 1.70, *P* = 1.15 × 10^–06^) and was unique to this age group. Comparing the three study groups, three *CDKAL1* SNPs (rs7756992, rs4712523, rs10946403) were found associated with T2D across ≥ 60, ≥ 65, and ≥ 70 control groups (Supplementary Table 3). Notably, for rs7756992, the ORs were 1.39 (≥ 60), 1.40 (≥ 65), and 1.49 (≥ 70), demonstrating a gradual increase in the association's strength with the age of control groups. Similarly, for rs4712523, the ORs were 1.36 (≥ 60), 1.39 (≥ 65), and 1.50 (≥ 70), indicating a consistent rise in association across these age groups. Moreover, for rs10946403, the ORs were 1.34 (≥ 60), 1.38 (≥ 65), and 1.52 (≥ 70), highlighting an increment in the strength of association as the control groups' age advanced. Alternative allele (G) was compared between T2D subjects and each of the specified age control groups (Supplementary Fig. 6). Two SNPs (rs4712523, rs7756992) were replicated in UK biobank, using individuals aged ≥ 65 without diabetes as controls (OR = 1.07; *P* = 2.12 × 10^–07^ and OR = 1.16; *P* = 9.28 × 10^–13^, respectively) (Supplementary Table 4). However, only rs7756992 showed replication when utilizing individuals aged ≥ 70 without diabetes as controls (OR = 1.14; *P* = 0.02) (Supplementary Fig. 7 and Supplementary Table 4).

### Interactions of CDKAL1 genetic variants and low HDL in age-related T2D risk

After establishing the link between the *CDKAL1* alternative alleles and the risk of T2D, the subsequent analysis delved into exploring the potential additive effect of low HDL when combined with these risk alleles. This investigation aimed to uncover whether the influence of low HDL further increased the risk of developing T2D in addition to the presence of these alleles (Table 2). The rs10946403*GG genotype increased T2D risk with OR = 1.62 (*P* < 0.001) for rs10946403*GG and 1.68 (*P* < 0.001) for rs10946403*GG combined with low HDL. This association persisted and intensified with age of control groups. In the ≥ 65 age group, ORs were 1.65 (*P* = 0.01) for rs10946403*GG and 1.71 (P < 0.001) for rs10946403*GG with low HDL, demonstrating consistent increments in T2D risk compared to controls. Notably, among individuals aged ≥ 70, the ORs were 2 (*P* < 0.001) for rs10946403*GG and 2.06 (P < 0.001) for rs10946403*GG combined with low HDL. Therefore, when combined with low HDL, the rs10946403 becomes more impactful as people get older in the control groups. Similar results were observed for rs4712523 and rs7756992.

**Table 2:** Impact of *CDKAL1* genetic variants and low HDL on T2D risk factors among older adults: Stepwise logistic regression and UK Biobank replication

**Study**	**SNPs ± Low HDL**	**OR**	**C.I.2.5%**	**C.I.97.5%**	***P*.value**
T2D.vs. noT2D aged ≥ 60	rs10946403GA	1.44	1.21	1.73	< 0.001
T2D.vs.noT2D aged ≥ 60	rs10946403GG	1.62	1.17	2.25	< 0.001
T2D.vs.noT2D aged ≥ 60	rs10946403GA.low HDL	1.48	1.23	1.78	< 0.001
T2D.vs.noT2D aged ≥ 60	rs10946403GG.low HDL	1.68	1.22	2.35	< 0.001
T2D.vs.noT2D aged ≥ 65	rs10946403GA	1.54	1.27	1.88	< 0.001
T2D.vs.noT2D aged ≥ 65	rs10946403GG	1.65	1.17	2.37	0.01
T2D.vs.noT2D aged ≥ 65	rs10946403GA.low HDL	1.57	1.28	1.92	< 0.001
T2D.vs.noT2D aged ≥ 65	rs10946403GG.low HDL	1.71	1.20	2.47	< 0.001
T2D.vs.noT2D aged ≥ 70	rs10946403GA	1.57	1.25	1.97	< 0.001
T2D.vs.noT2D aged ≥ 70	rs10946403GG	2.00	1.32	3.14	< 0.001
T2D.vs.noT2D aged ≥ 70	rs10946403GA.low HDL	1.60	1.27	2.01	< 0.001
T2D.vs.noT2D aged ≥ 70	rs10946403GG.low HDL	2.06	1.34	3.24	< 0.001
T2D.vs.noT2D aged ≥ 60	rs4712523GA	1.42	1.18	1.70	< 0.001
T2D.vs.noT2D aged ≥ 60	rs4712523GG	1.74	1.30	2.33	< 0.001
T2D.vs.noT2D aged ≥ 60	rs4712523GA.low HDL	1.45	1.20	1.74	< 0.001
T2D.vs.noT2D aged ≥ 60	rs4712523GG.low HDL	1.78	1.33	2.40	< 0.001
T2D.vs.noT2D aged ≥ 65	rs4712523GA	1.45	1.19	1.77	< 0.001
T2D.vs.noT2D aged ≥ 65	rs4712523GG	1.77	1.29	2.45	< 0.001
T2D.vs.noT2D aged ≥ 65	rs4712523GA.low HDL	1.47	1.20	1.79	< 0.001
T2D.vs.noT2D aged ≥ 65	rs4712523GG.low HDL	1.81	1.31	2.51	< 0.001
T2D.vs.noT2D aged ≥ 70	rs4712523GA	1.47	1.17	1.84	< 0.001
T2D.vs.noT2D aged ≥ 70	rs4712523GG	2.08	1.43	3.10	< 0.001
T2D.vs.noT2D aged ≥ 70	rs4712523GA.low HDL	1.49	1.18	1.87	< 0.001
T2D.vs.noT2D aged ≥ 70	rs4712523GG.low HDL	2.11	1.44	3.16	< 0.001
T2D.vs.noT2D aged ≥ 60	rs7756992GA	1.44	1.20	1.73	< 0.001
T2D.vs.noT2D aged ≥ 60	rs7756992GG	1.81	1.34	2.46	< 0.001
T2D.vs.noT2D aged ≥ 60	rs7756992GA.low HDL	1.49	1.24	1.79	< 0.001
T2D.vs.noT2D aged ≥ 60	rs7756992GG.low HDL	1.89	1.39	2.58	< 0.001
T2D.vs.noT2D aged ≥ 65	rs7756992GA	1.46	1.20	1.78	< 0.001
T2D.vs.noT2D aged ≥ 65	rs7756992GG	1.83	1.32	2.57	< 0.001
T2D.vs.noT2D aged ≥ 65	rs7756992GA.low HDL	1.49	1.22	1.82	< 0.001
T2D.vs.noT2D aged ≥ 65	rs7756992GG.low HDL	1.90	1.36	2.68	< 0.001
T2D.vs.noT2D aged ≥ 70	rs7756992GA	1.44	1.15	1.81	< 0.001
T2D.vs.noT2D aged ≥ 70	rs7756992GG	2.12	1.43	3.22	< 0.001
T2D.vs.noT2D aged ≥ 70	rs7756992GA.low HDL	1.46	1.17	1.84	< 0.001
T2D.vs.noT2D aged ≥ 70	rs7756992GG.low HDL	2.20	1.47	3.35	< 0.001
**Replication in the UK Biobank**					
**Study**	**SNPs**	**OR**	**C.I.2.5%**	**C.I.97.5%**	***P*.value**
T2D.vs.noT2D aged ≥ 65	rs4712523GA	1.04	1.00	1.08	0.05
T2D.vs.noT2D aged ≥ 65	rs4712523GG	1.18	1.11	1.26	< 0.001
T2D.vs.noT2D aged ≥ 65	rs4712523GA.low HDL	1.04	1.00	1.08	0.07
T2D.vs.noT2D aged ≥ 65	rs4712523GG.low HDL	1.19	1.11	1.27	< 0.001
T2D.vs.noT2D aged ≥ 70	rs4712523GA	0.95	0.81	1.11	0.53
T2D.vs.noT2D aged ≥ 70	rs4712523GG	1.38	1.04	1.85	0.03
T2D.vs.noT2D aged ≥ 70	rs4712523GA.low HDL	0.94	0.81	1.11	0.48
T2D.vs.noT2D aged ≥ 70	rs4712523GG.low HDL	1.38	1.04	1.86	0.03
T2D.vs.noT2D aged ≥ 65	rs7756992GA	1.06	1.02	1.10	< 0.001
T2D.vs.noT2D aged ≥ 65	rs7756992GG	1.28	1.20	1.38	< 0.001
T2D.vs.noT2D aged ≥ 65	rs7756992GA.low HDL	1.06	1.02	1.10	0.01
T2D.vs.noT2D aged ≥ 65	rs7756992GG.low HDL	1.29	1.20	1.38	< 0.001
T2D.vs.noT2D aged ≥ 70	rs7756992GA	1.05	0.90	1.24	0.52
T2D.vs.noT2D aged ≥ 70	rs7756992GG	1.46	1.07	2.03	0.02
T2D.vs.noT2D aged ≥ 70	rs7756992GA.low HDL	1.05	0.90	1.23	0.55
T2D.vs.noT2D aged ≥ 70	rs7756992GG.low HDL	1.46	1.07	2.03	0.02

*T2D* Type 2 Diabetes, *SNPs* single nucleotide polymorphisms, *OR* odd ratio, *C.I.2.5%* lower bound of the 95% confidence interval for the odds ratio, *C.I.97.5%* upper bound of the 95% confidence interval for the odds ratio, *G* alternative allele, *A* reference allele, *GG* homozygous alternative genotype, *GA* heterozygous genotype, *HDL* high-density lipoprotein, *Low HDL* HDL < 40 mg/dl

## Discussion

### Age-related impact of T2D susceptibility variants and HDL levels on T2D risk

The study investigated the association between T2D susceptibility variants and HDL levels on the risk of developing T2D in the older adults. The uniqueness of this study lies in the stratification of control groups based on age [three age ranges (≥ 60, ≥ 65, and ≥ 70 years)]. This study explores the significant associations between T2D and Fx of T2D, notably strengthening with age among control groups. In addition, a considerable association was detected between T2D and low HDL cholesterol levels, especially evident in older control groups. These findings suggest a potential age-related impact on T2D susceptibility and highlight the importance of monitoring HDL levels with age. In addition, T2D susceptibility variants are associated with T2D and this association increases significantly with age of control groups.

### SNPs implicated in T2D susceptibility in older adults through GWAS

GWAS identified specific genetic variants associated with T2D. Herein, three *CDKAL1* gene variants (rs7756992, rs4712523 and rs10946403) were associated with T2D, with a higher association with the age of control groups. *CDKAL1*, functioning as a tRNA modifier from the methylthiotransferase family, plays a crucial role in post-translational modification of insulin following glucose stimulation. Two variants, rs7756992 and rs4712523, were replicated in the UK Biobank. These variants are reportedly associated with increased T2D risk [37–39]. Interestingly, the *CDKAL1* gene variant rs11967068 showed a robust association with T2D solely when taking individuals aged ≥ 70 years as controls. This previously unreported link might be age related and may be specific to T2D development in older adults suggesting that additional factors play a role in modulating its susceptibility impact. Further research is required to elucidate the factors influencing this association. *TCF7L2* variants (rs7903146 and rs7901695) were positively associated with T2D, primarily in the youngest age control group. The rs7903146 impairs glucose tolerance through glucose stimulated insulin secretion and sensitivity of the β cell to incretins, rather than actual insulin action [40]. The relationship between this SNP and T2D has been investigated across various ethnicities [38]. The absence of this SNP in the other specified age controls might be also stemming from sample size limitations. Larger samples, like the ≥ 70 in the UK Biobank, replicated this association. Two SNPs in *ATP2B2* (ATPase Plasma Membrane Ca^2^^+^ Transporting 2) and *IGFN1* (immunoglobulin like and fibronectin type III domain containing 1) were associated with T2D in ≥ 70 controls. The rs7629204 in *ATP2B2* was positively associated with T2D which could suggest a role in irregular calcium homeostasis and signaling associated with both T2D and ageing. The rs3738270 of *IGFN1* was negatively associated with T2D in ≥ 65 and ≥ 70 controls and this association increased with the age of the control groups. This suggests a role for *IGFN1* in lowering T2D risk. *IGFN* 1 encodes a protein characterized by immunoglobulin like and fibronectin type III domain. This protein is essential for myoblast fusion and differentiation and exhibits predominant expression in skeletal muscle [41]. The reduction in both skeletal muscle mass and function stands as a primary contributor to frailty in older adults [42]. Decreased expression of *IGFN1* is associated with muscle aging. Conversely, elevated expression of *IGFN1* has been associated with enhanced performance. This is evident from studies with aged muscle [41]. Multiple research have highlighted the connection between muscle parameters and T2D [43]. These studies consistently report a reduction in muscle quality (strength) and mass among individuals with T2D compared with control groups. Specifically, older adults with diabetes exhibit a decline in muscle mass compared to their age-matched counterparts [44, 45]. *IGFN1*’s “protective” effect through its negative association in T2D of the older age group suggests a possible role in counteracting the functional decline associated with age [46] which could be beneficial in managing or preventing T2D. This may be due to its importance in muscle health which is critical in preventing frailty and maintaining metabolic function with age.

### Low HDL cholesterol and genetic variants in modulating T2D risk

Furthermore, this study explored the combined effect of low HDL and the detected genetic variants on T2D risk. Certain SNPs (rs10946403, rs4712523, rs7756992) had a stronger effect on T2D risk when combined with low HDL cholesterol levels, especially in older control groups. This suggests that as individuals age, with the absence of these genetic variants and the presence of high HDL, the risk of developing T2D decreases. This triad protective mechanism, involving age, HDL and genetics, could serve as a valuable insight for identifying individuals who are at a reduced risk of T2D and potentially inform targeted preventive strategies. Profound HDL dysfunction is typical in T2D, linked to hypertriglyceridemia, hyperglycemia, inflammation and oxidative stress [47]. Healthy lifestyle have been associated with reduced diabetes complications. Studies have linked lifestyle behaviors, lipid profiles, liver and renal function biomarkers, blood pressure indices, improvements in glycemic control, and systemic inflammatory markers. Furthermore, lifestyle modifications such as healthy diet, physical activity and weight loss can improve liver and renal function, the lipid profile and endothelial dysfunction and can reduce inflammation [47, 48], highlighting lifestyle’s role in diabetes risk alongside genetic predisposition [49, 50]. The combination of factors, including the presence of specific genetic variants, age, and adherence to a healthy lifestyle, forms a comprehensive framework for understanding and managing the risk of T2D.

### Strengths and limitations

The strength and novelty of this study lies in the examination of genetic variants and T2D risk across ages combined with HDL levels. While the sample size numbers are lower with ageing, the results were replicated in the UK Biobank. However, limitations include potential sample biases, limited generalizability and the incomplete explanatory power of GWAS studies for T2D heritability. The sample composition, predominantly older adults, might introduce biases due to age-related variations in genetic susceptibility and lifestyle factors. The generalizability of the findings might be limited due to the population’s specific characteristics and the use of a specific dataset. Moreover, GWAS limits the ability to establish causation between identified factors and T2D, emphasizing the need for functional studies.

## Conclusions

In conclusion, stratifying control group by age helps identify protective factors to older age groups. Combining genetics, age and HDL levels is vital in assessing T2D risk in the older adults. High HDL levels without T2D susceptibility alleles may have the lowest risk in older individuals. Identifying protective factors in older adults could potentially guide interventions for T2D prevention and healthy aging [51]. Exploring HDL's role in various metabolic processes associated with aging may unravel its broader impact beyond T2D susceptibility. The identification of specific genetic variants associated with T2D susceptibility, particularly when combined with low HDL levels, offers a significant opportunity for personalized risk assessment. This insight enables healthcare providers to stratify patients based on genetic predisposition and HDL levels, facilitating tailored interventions and preventive measures.

### Future perspectives

Future research could focus on how age-related changes in HDL composition and function may influence the development of T2D in older individuals. Considering the capacity of lipid-related processes to influence both lifespan and healthspan in model organisms, they are emerging as a promising elixir and clinical intervention to enhance human lifespan [52]. By implementing targeted strategies to manage HDL levels and address genetic susceptibilities, clinicians can potentially mitigate T2D risk in this aging population. These insights emphasize the importance of personalized care and highlight avenues for the development of more effective preventive strategies in clinical practice.

### Supplementary Information

**Additional file 1:** **Supplementary Figure 1.** Flowchart of Study Methodology and Analysis Steps. T2D: Type 2 Diabetes. **Supplementary Figure 2.** (a) Fx T2D, (b) HDL levels, and (c) low and high HDL according to T2D status and age. **Supplementary Figure 3.** HDL levels according to T2D status and age using the UK biobank. **Supplementary Figure 4.** (a) Manhattan plot for the genome-wide association analysis with T2D taking non-diabetic aged ≥60 as controls. (b) Quantile-Quantile (Q-Q) plot of the GWAS results showing the distribution of *p*-values, plotted against the expected distribution. (c) Regional association plots for the loci associated with T2D among elderlies in the Lebanese population. **Supplementary Figure 5.** (a) Manhattan plot for the genome-wide association analysis with T2D taking non-diabetic aged ≥65 as controls. (b) Quantile-Quantile (Q-Q) plot of the GWAS results showing the distribution of p-values, plotted against the expected distribution. (c) Regional association plots for the loci associated with T2D among elderlies in the Lebanese population. **Supplementary Figure 6.** Minor allele frequencies of three *CDKAL1* SNPs according to T2D status and age. **Supplementary Figure 7.** Replication of the Manhattan plot for the genome-wide association analysis with T2D taking non-diabetic aged ≥70 as controls using the UK Biobank.

**Additional file 2:** **Supplementary Table 1.** Comparison of categorical variables between patients with diabetes and subjects without diabetes of different age groups. **Supplementary Table 2.** Odds ratio of the genetic loci associated with T2D using individuals without diabetes (aged ≥60) as controls derived from the genome-wide association analysis. **Supplementary Table 3.** Comparison of the odds ratio of the *CDKAL1* SNPs and T2D among older adults derived from the genome-wide association analysis. **Supplementary Table 4.** Replication of the association between the risk loci and T2D taking individuals without diabetes aged ≥65 or aged ≥70 as controls in the UK Biobank.

## Data Availability

The datasets used and/or analysed during the current study are available from the corresponding author on reasonable request.

## Abbreviations

T2D: Type 2 Diabetes
HDL: High Density Lipoprotein
GWAS: Genome Wide Association Study
SNPs: Single nucleotide polymorphism
Fx: Family history
OR: Odd ratio
*P*: *p*-Value
Q-Q: Quantile–quantile
MAF: Minor allele frequency
IGFN1: Immunoglobulin Like And Fibronectin Type III Domain Containing 1
ATP2B2: ATPase Plasma Membrane Ca2 + Transporting 2
TCF7L2: Transcription factor 7-like 2
CDKAL1: Cdk5 regulatory associated protein 1-like 1
tRNA: Transfer Ribonucleic Acid
EUR: Europe
US: United States of America
UK: United kingdom
GRINA: Glutamate Receptor, Ionotropic, N-Methyl D-Aspartate-Associated Protein 1
GPR146: G Protein-Coupled Receptor 146
VLDL: Very Low Density Lipoprotein
LDL: Low Density Lipoprotein
BMI: Body mass index
CRP: C-reactive protein
