# Supplementary material for: HDL levels modulate the impact of type 2 diabetes susceptibility alleles in older adults
Source: Lipids Health Dis. 2024 Feb 22;23:56. doi: 10.1186/s12944-024-02039-7 (PMC10882764; doi:10.1186/s12944-024-02039-7)
Supplement: Supplementary file 1 — Additional file 1: Supplementary Figure 1. Flowchart of Study Methodology and Analysis Steps. T2D: Type 2 Diabetes. Supplementary Figure 2. (a) Fx T2D, (b) HDL levels, and (c) low and high HDL according to T2D status and age. Supplementary Figure 3. HDL levels according to T2D status and age using the UK biobank. Supplementary Figure 4. (a) Manhattan plot for the genome-wide association analysis with T2D taking non-diabetic aged ≥60 as controls. (b) Quantile-Quantile (Q-Q) plot of the GWAS results showing the distribution of p-values, plotted against the expected distribution. (c) Regional association plots for the loci associated with T2D among elderlies in the Lebanese population. Supplementary Figure 5. (a) Manhattan plot for the genome-wide association analysis with T2D taking non-diabetic aged ≥65 as controls. (b) Quantile-Quantile (Q-Q) plot of the GWAS results showing the distribution of p-values, plotted against the expected distribution. (c) Regional association plots for the loci associated with T2D among elderlies in the Lebanese population. Supplementary Figure 6. Minor allele frequencies of three CDKAL1 SNPs according to T2D status and age. Supplementary Figure 7. Replication of the Manhattan plot for the genome-wide association analysis with T2D taking non-diabetic aged ≥70 as controls using the UK Biobank. [file 12944_2024_2039_MOESM1_ESM.pdf]

Supplementary Figure 1. Flowchart of Study Methodology and Analysis Steps. T2D: Type 2 diabetes.

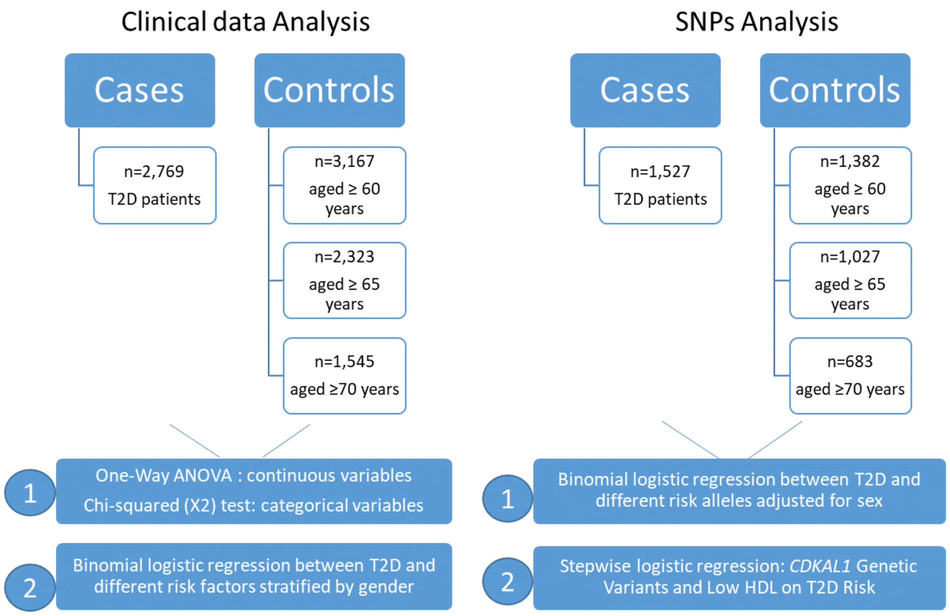

Supplementary Figure 2. (a) Fx T2D, **(b)** HDL levels, and **(c)** low and high HDL according to T2D status and age.

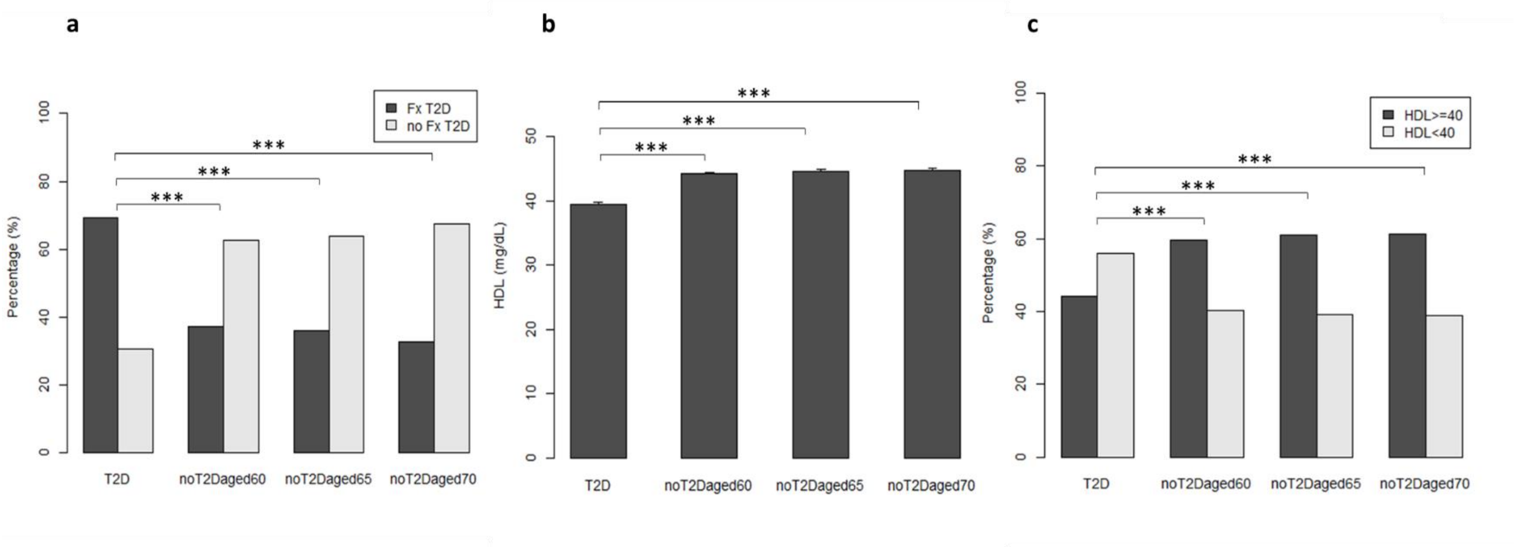

T2D: Type 2 diabetes. Fx: family history. (a) Data are count of Fx T2D among the groups. (b) Data are mean values of HDL in mg/dl among the groups. (c) Data are count of low hdl (HDL <40 mg/dl) or high HDL (HDL ≥ 40 mg/dl) among the groups. ***: p value <0.01.

Supplementary Figure 3. HDL levels according to T2D status and age using the UK biobank.

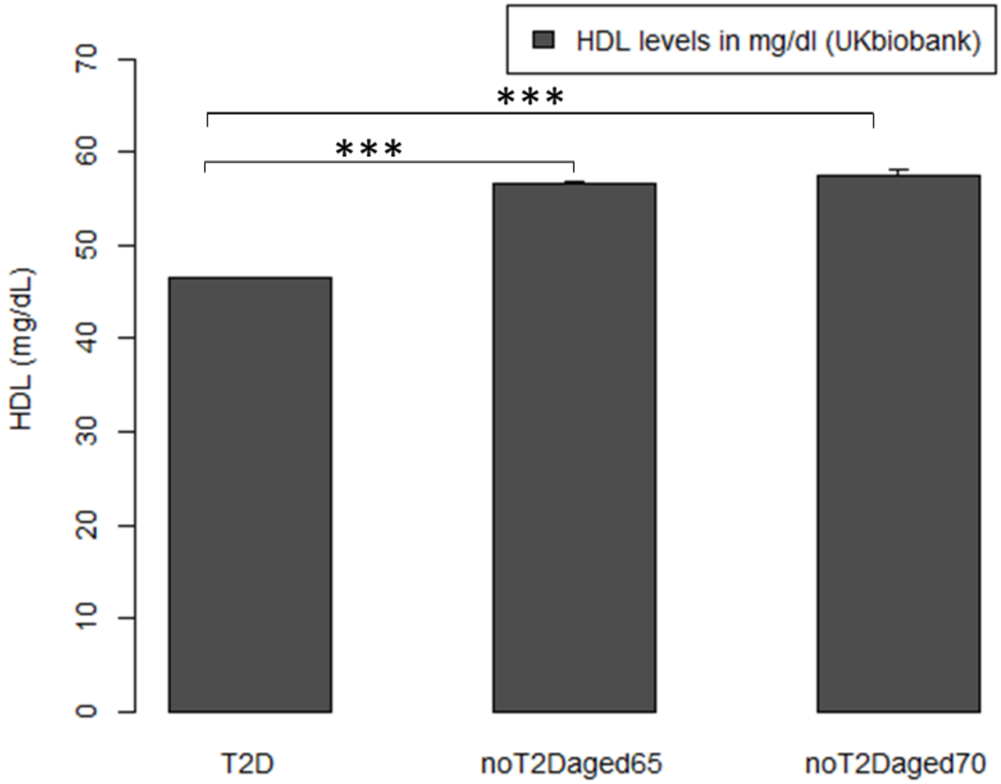

T2D: Type 2 diabetes. Data are mean values of HDL in mg/dl among the groups. ***: p value <0.01.

Supplementary Figure 4 (**a**) Manhattan plot for the genome-wide association analysis with T2D taking non-diabetic aged ≥60 as controls. (**b**) Quantile-Quantile (Q-Q) plot of the GWAS results showing the distribution of p-values, plotted against the expected distribution. (**c**) Regional association plots for the loci associated with T2D among elderlies in the Lebanese population.

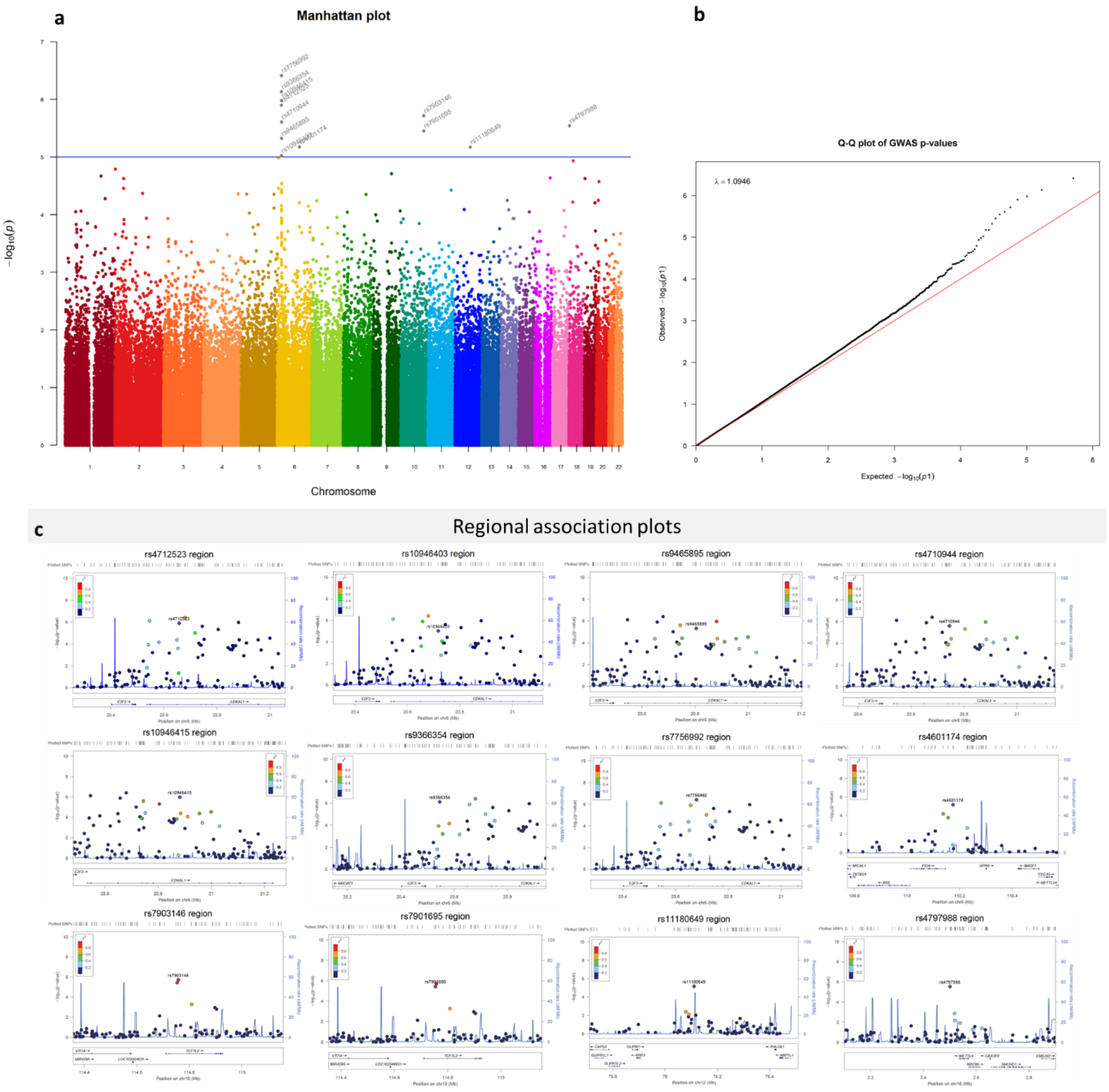

GWAS: genome-wide association study. T2D: Type 2 diabetes. (**a**) X-axis shows chromosomal positions. Y axis represents the $-\log_{10}$(P-value obtained) by logistic regression analysis (additive model) after adjusting for sex. The horizontal solid blue line indicates the suggestive genome-wide threshold of P = 1 x 10^{-5}. Each point denotes a Single nucleotide polymorphism (SNP), SNPs with significance P < 1 x 10^{-5} are shown above the suggestive significance line in blue. The annotated SNPs in black are targeted T2D susceptibility SNPs. (**b**) This plot shows the association between each tested SNP and the observed -log10 p values, plotted on the vertical axis, compared to the expected –log10 p values under the null hypothesis. Each dot on the plot represents a SNP. The genomic control ratio (l) was 1.0946, which indicates that there is no strong effect of systematic error, such as population stratification. (**c**) Correlations between the SNPs with the lowest P value from GWAS (depicted in purple) and nearby SNPs within a 400 kb region. The r^2 values of the Linkage Disequilibrium (LD) heat map is based on the hg19/1000 genomes Nov 2014 EUR reference set.

Supplementary Figure 5 (**a**) Manhattan plot for the genome-wide association analysis with T2D taking non-diabetic aged ≥65 as controls. (**b**) Quantile-Quantile (Q-Q) plot of the GWAS results showing the distribution of p-values, plotted against the expected distribution. (**c**) Regional association plots for the loci associated with T2D among elderlies in the Lebanese population.

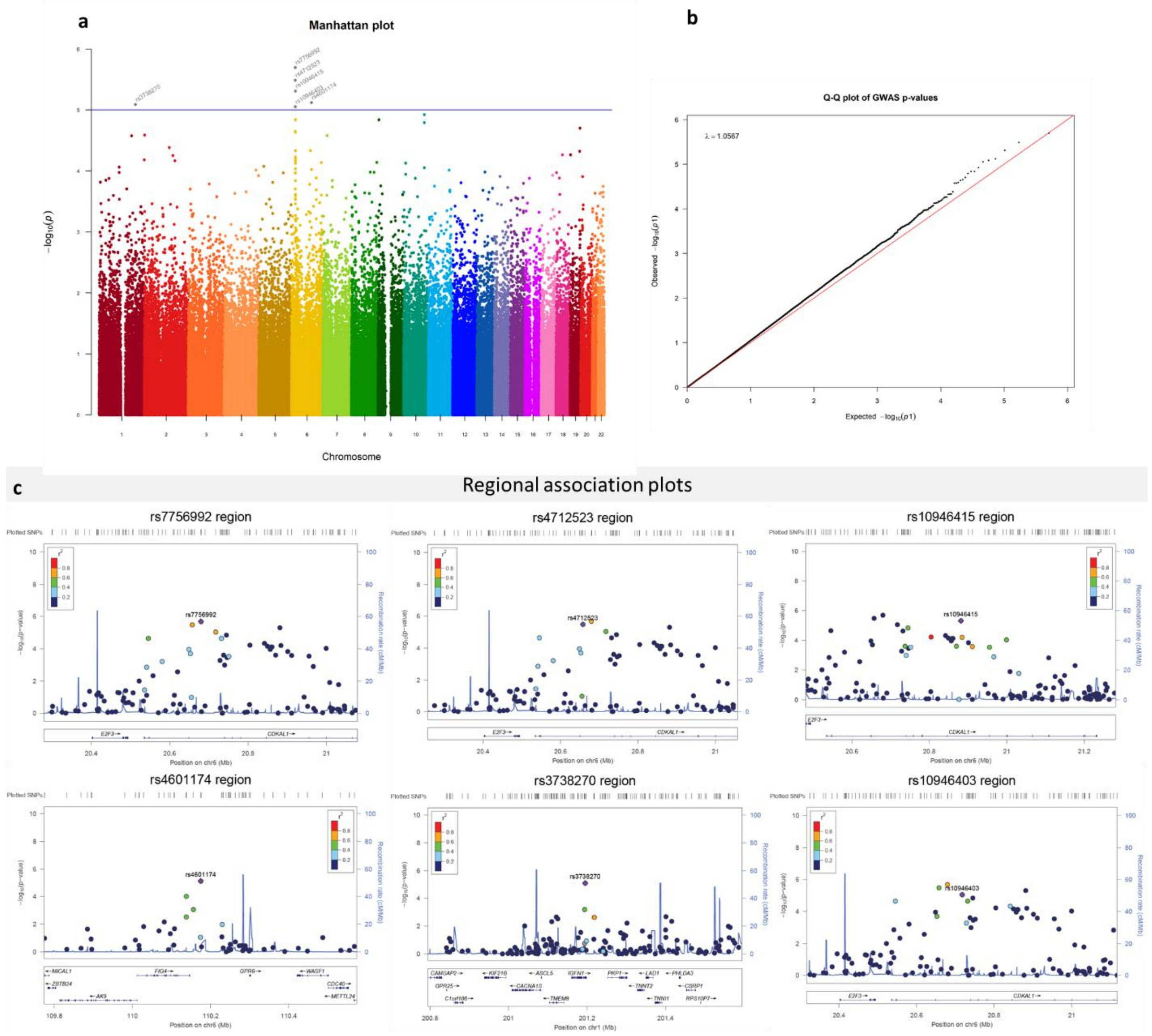

GWAS: genome-wide association study. T2D: Type 2 diabetes. (**a**) X-axis shows chromosomal positions. Y axis represents the $-\log_{10}$(P-value obtained) by logistic regression analysis (additive model) after adjusting for sex. The horizontal solid blue line indicates the suggestive genome-wide threshold of $P = 1 \times 10^{-5}$. Each point denotes a Single nucleotide polymorphism (SNP), SNPs with significance $P < 1 \times 10^{-5}$ (red points) are shown above the suggestive significance blue line. The annotated SNPs in black are targeted T2D susceptibility SNPs. (**b**) This plot shows the association between each tested SNP and the observed -log10 p values, plotted on the vertical axis, compared to the expected –log10 p values under the null hypothesis. Each dot on the plot represents a SNP. The genomic control ratio (l) was 1.0567, which indicates that there is no strong effect of systematic error, such as population stratification. (**c**) Correlations between the SNPs with the lowest P value from GWAS (depicted in purple) and nearby SNPs within a 400 kb region. The r^2 values of the Linkage Disequilibrium (LD) heat map is based on the hg19/1000 genomes Nov 2014 EUR reference set.

Supplementary Figure 6. Minor allele frequencies of three *CDKAL1* SNPs according to T2D status and age.

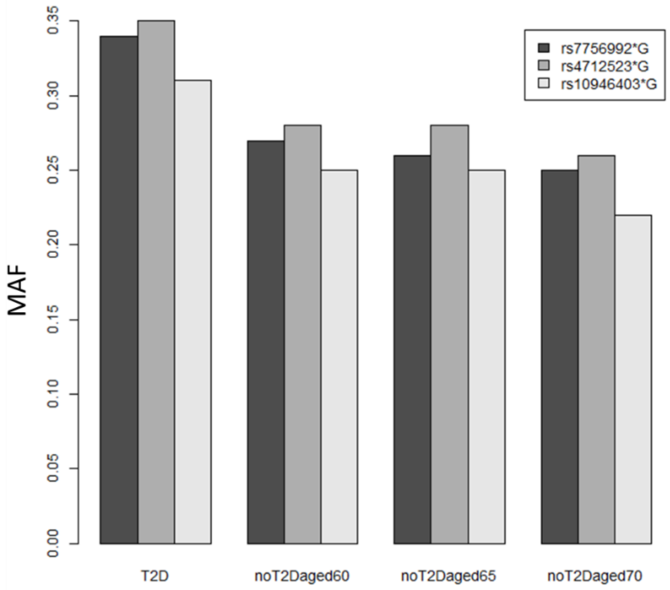

T2D: Type 2 diabetes. MAF: minor allele frequency. G: derived allele.

Supplementary Figure 7. Replication of the Manhattan plot for the genome-wide association analysis with T2D taking non-diabetic aged ≥70 as controls using the UK Biobank.

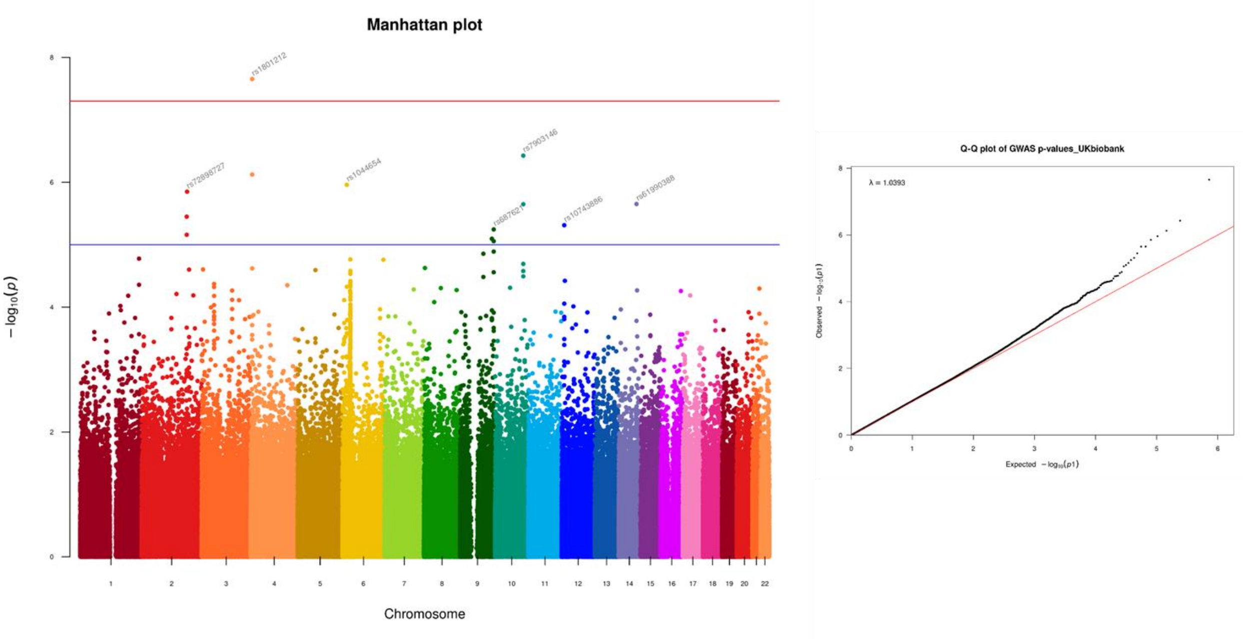

GWAS: genome-wide association study. T2D: Type 2 diabetes. (a) X-axis shows chromosomal positions. Y axis represents the $-\log_{10}$(P-value obtained) by logistic regression analysis (additive model). The horizontal solid blue line indicates the suggestive genome-wide threshold of P = 1 x 10^{-5}. Each point denotes a Single nucleotide polymorphism (SNP). SNPs with significance P < 5 × 10^{-8} are shown above the genome-wide significance in red.
