# Supplementary material for: HDL levels modulate the impact of type 2 diabetes susceptibility alleles in older adults
Source: Lipids Health Dis. 2024 Feb 22;23:56. doi: 10.1186/s12944-024-02039-7 (PMC10882764; doi:10.1186/s12944-024-02039-7)
Supplement: Supplementary file 2 — Additional file 2: Supplementary Table 1. Comparison of categorical variables between patients with diabetes and subjects without diabetes of different age groups. Supplementary Table 2. Odds ratio of the genetic loci associated with T2D using individuals without diabetes (aged ≥60) as controls derived from the genome-wide association analysis. Supplementary Table 3. Comparison of the odds ratio of the CDKAL1 SNPs and T2D among older adults derived from the genome-wide association analysis. Supplementary Table 4. Replication of the association between the risk loci and T2D taking individuals without diabetes aged ≥65 or aged ≥70 as controls in the UK Biobank. [file 12944_2024_2039_MOESM2_ESM.docx]

**Supplementary Table 1**. Comparison of categorical variables between patients with diabetes and subjects without diabetes of different age groups.

|  | T2D | noT2D ≥ 60 years | noT2D ≥ 65 years | noT2D ≥ 70 years | P1 | P2 | P3 |
| --- | --- | --- | --- | --- | --- | --- | --- |
| N | 2769 | 3167 | 2323 | 1545 |  |  |  |
| Sex |  |  |  |  | 0.67 | 0.58 | 0.32 |
| Female | 1084 (39.2%) | 1223 (38.6%) | 927 (39.9%) | 629 (40.7%) |  |  |  |
| Male | 1684 (60.8%) | 1943 (61.4%) | 1395 (60.1%) | 916 (59.3%) |  |  |  |
| BMI≥25 |  |  |  |  | < 0.01 | < 0.01 | < 0.01 |
| NO | 430 (16.3%) | 722 (24.1%) | 545 (24.9%) | 377 (26.0%) |  |  |  |
| YES | 2210 (83.7%) | 2280 (75.9%) | 1643 (75.1%) | 1071 (74.0%) |  |  |  |
| HDL<40 |  |  |  |  | < 0.01 | < 0.01 | < 0.01 |
| NO | 1047 (44.1%) | 1545 (59.7%) | 1153 (60.9%) | 763 (61.2%) |  |  |  |
| YES | 1329 (55.9%) | 1042 (40.3%) | 741 (39.1%) | 484 (38.8%) |  |  |  |
| CVD |  |  |  |  | < 0.01 | 0.08 | 0.49 |
| NO | 795 (28.9%) | 1034 (32.9%) | 717 (31.2%) | 457 (29.9%) |  |  |  |
| YES | 1958 (71.1%) | 2107 (67.1%) | 1584 (68.8%) | 1073 (70.1%) |  |  |  |
| Hypertension |  |  |  |  | < 0.01 | < 0.01 | < 0.01 |
| NO | 775 (28.0%) | 1173 (37.0%) | 808 (34.8%) | 503 (32.6%) |  |  |  |
| YES | 1993 (72.0%) | 1993 (63.0%) | 1514 (65.2%) | 1041 (67.4%) |  |  |  |
| Hyperlipidemia |  |  |  |  | < 0.01 | < 0.01 | < 0.01 |
| NO | 1148 (41.5%) | 1791 (56.7%) | 1329 (57.4%) | 912 (59.2%) |  |  |  |
| YES | 1616 (58.5%) | 1367 (43.3%) | 987 (42.6%) | 628 (40.8%) |  |  |  |
| Fx CVD |  |  |  |  | < 0.01 | < 0.01 | < 0.01 |
| NO | 1185 (43.1%) | 1515 (48.4%) | 1153 (50.3%) | 810 (53.3%) |  |  |  |
| YES | 1562 (56.9%) | 1614 (51.6%) | 1137 (49.7%) | 709 (46.7%) |  |  |  |
| Fx Hypertension |  |  |  |  | < 0.01 | < 0.01 | < 0.01 |
| NO | 1183 (43.1%) | 1587 (50.7%) | 1210 (52.9%) | 819 (54.0%) |  |  |  |
| YES | 1559 (56.9%) | 1542 (49.3%) | 1079 (47.1%) | 698 (46.0%) |  |  |  |
| Fx Hyperlipidemia |  |  |  |  | < 0.01 | < 0.01 | < 0.01 |
| NO | 1769 (64.4%) | 2260 (72.0%) | 1667 (72.3%) | 1134 (73.9%) |  |  |  |
| YES | 976 (35.6%) | 880 (28.0%) | 639 (27.7%) | 400 (26.1%) |  |  |  |
| Fx T2D |  |  |  |  | < 0.01 | < 0.01 | < 0.01 |
| NO | 842 (30.6%) | 1965 (62.7%) | 1466 (63.9%) | 1026 (67.5%) |  |  |  |
| YES | 1907 (69.4%) | 1167 (37.3%) | 827 (36.1%) | 495 (32.5%) |  |  |  |

Data are count followed by percentage. The *p*-value is generated using Chi-Squared Test. P-value is significant if p.value < 0.05. T2D: Type 2 diabetes. CVD: Cardiovascular disease. Fx: Family history. BMI: Body Mass Index. P1: p.value between T2D and noT2D ≥ 60 years. P2: p.value between T2D and noT2D ≥ 65 years. P3: p.value between T2D and noT2D ≥ 70 years.

**Supplementary Table 2**. Odds ratio of the genetic loci associated with T2D using individuals without diabetes (aged≥60) as controls derived from the genome-wide association analysis.

| **Gene** | **CHR** | **SNP** | **BP** | **A1** | **A2** | **OR** | **L95** | **U95** | **P** |
| --- | --- | --- | --- | --- | --- | --- | --- | --- | --- |
| **T2D vs. no T2D aged≥60** | | | | | | | | | |
| CDKAL1 | 6 | rs7756992 | 20679709 | G | A | 1.39 | 1.23 | 1.58 | 3.86E-07 |
| CDKAL1 | 6 | rs9366354 | 20545468 | G | A | 1.34 | 1.2 | 1.51 | 7.36E-07 |
| CDKAL1 | 6 | rs10946415 | 20881386 | T | C | 0.71 | 0.62 | 0.81 | 1.05E-06 |
| CDKAL1 | 6 | rs4712523 | 20657564 | G | A | 1.36 | 1.2 | 1.55 | 1.26E-06 |
| TCF7L2 | 10 | rs7903146 | 114758349 | T | C | 1.35 | 1.19 | 1.52 | 1.92E-06 |
| CDKAL1 | 6 | rs4710944 | 20744727 | T | C | 0.73 | 0.64 | 0.83 | 2.46E-06 |
| NA | 18 | rs4797988 | 2500382 | T | C | 0.74 | 0.66 | 0.84 | 2.87E-06 |
| TCF7L2 | 10 | rs7901695 | 114754088 | C | T | 1.34 | 1.18 | 1.51 | 3.54E-06 |
| CDKAL1 | 6 | rs9465895 | 20804563 | C | T | 0.73 | 0.63 | 0.83 | 4.76E-06 |
| NA | 6 | rs4601174 | 110174645 | A | C | 1.31 | 1.17 | 1.48 | 6.68E-06 |
| LOC105369844 | 12 | rs11180649 | 76110751 | G | A | 0.71 | 0.61 | 0.82 | 6.70E-06 |
| CDKAL1 | 6 | rs10946403 | 20717404 | G | A | 1.34 | 1.18 | 1.53 | 9.48E-06 |
| **T2D vs. no T2D aged≥65** | | | | | | | | | |
| CDKAL1 | 6 | rs7756992 | 20679709 | G | A | 1.4 | 1.22 | 1.61 | 2.01E-06 |
| CDKAL1 | 6 | rs4712523 | 20657564 | G | A | 1.39 | 1.21 | 1.59 | 3.24E-06 |
| CDKAL1 | 6 | rs10946415 | 20881386 | T | C | 0.71 | 0.61 | 0.82 | 4.89E-06 |
| NA | 6 | rs4601174 | 110174645 | A | C | 1.34 | 1.18 | 1.53 | 7.55E-06 |
| IGFN1 | 1 | rs3738270 | 201195119 | T | C | 0.74 | 0.65 | 0.85 | 8.16E-06 |
| CDKAL1 | 6 | rs10946403 | 20717404 | G | A | 1.38 | 1.2 | 1.6 | 8.86E-06 |
| **T2D vs. no T2D aged≥70** | | | | | | | | | |
| CDKAL1 | 6 | rs11967068 | 20841593 | C | T | 1.84 | 1.45 | 2.33 | 4.03E-07 |
| NA | 1 | rs7554231 | 1.11E+08 | G | A | 1.57 | 1.31 | 1.89 | 9.18E-07 |
| CDKAL1 | 6 | rs4712523 | 20657564 | G | A | 1.5 | 1.27 | 1.76 | 9.53E-07 |
| CDKAL1 | 6 | rs10946403 | 20717404 | G | A | 1.52 | 1.29 | 1.81 | 1.14E-06 |
| ATP2B2 | 3 | rs7629204 | 10641274 | G | A | 1.7 | 1.37 | 2.1 | 1.15E-06 |
| CDKAL1 | 6 | rs7756992 | 20679709 | G | A | 1.49 | 1.27 | 1.76 | 1.51E-06 |
| CDKAL1 | 6 | rs16901585 | 20861152 | A | G | 1.55 | 1.3 | 1.86 | 1.82E-06 |
| IGFN1 | 1 | rs3738270 | 201195119 | T | C | 0.69 | 0.59 | 0.8 | 1.86E-06 |
| NA | 10 | rs315580 | 36484336 | C | T | 1.84 | 1.43 | 2.36 | 2.21E-06 |
| CDKAL1 | 6 | rs7753271 | 20849412 | T | C | 1.57 | 1.3 | 1.89 | 2.40E-06 |
| CDKAL1 | 6 | rs9366363 | 20858852 | G | T | 0.68 | 0.57 | 0.8 | 4.95E-06 |

SNPs: single nucleotide polymorphisms. CHR: chromosome. BP: Base Pair. A1: Alternate allele. A2: reference allele. P: P-value. OR: odd ratio. T2D: type 2 diabetes. L95 and U95: lower and upper limits of the 95% confidence interval for the OR estimate. NA: not available.

**Supplementary Table 3**. Comparison of the odds ratio of the *CDKAL1* SNPs and T2D among older adults derived from the genome-wide association analysis.

| Gene |  | CHR | SNP | BP | A1 | A2 | F_A | F_U | OR | L95 | U95 | P |
| --- | --- | --- | --- | --- | --- | --- | --- | --- | --- | --- | --- | --- |
| CDKAL1 | T2D vs. no T2D aged≥60 | 6 | rs7756992 | 20679709 | G | A | 0.34 | 0.27 | 1.39 | 1.23 | 1.58 | 3.86E-07 |
| CDKAL1 | T2D vs. no T2D aged≥65 | 6 | rs7756992 | 20679709 | G | A | 0.34 | 0.26 | 1.4 | 1.22 | 1.61 | 2.01E-06 |
| CDKAL1 | T2D vs. no T2D aged≥70 | 6 | rs7756992 | 20679709 | G | A | 0.34 | 0.25 | 1.49 | 1.27 | 1.76 | 1.51E-06 |
| CDKAL1 | T2D vs. no T2D aged≥60 | 6 | rs4712523 | 20657564 | G | A | 0.35 | 0.28 | 1.36 | 1.2 | 1.55 | 1.26E-06 |
| CDKAL1 | T2D vs. no T2D aged≥65 | 6 | rs4712523 | 20657564 | G | A | 0.35 | 0.28 | 1.39 | 1.21 | 1.59 | 3.24E-06 |
| CDKAL1 | T2D vs. no T2D aged≥70 | 6 | rs4712523 | 20657564 | G | A | 0.35 | 0.26 | 1.5 | 1.27 | 1.76 | 9.53E-07 |
| CDKAL1 | T2D vs. no T2D aged≥60 | 6 | rs10946403 | 20717404 | G | A | 0.31 | 0.25 | 1.34 | 1.18 | 1.53 | 9.48E-06 |
| CDKAL1 | T2D vs. no T2D aged≥65 | 6 | rs10946403 | 20717404 | G | A | 0.31 | 0.25 | 1.38 | 1.2 | 1.6 | 8.86E-06 |
| CDKAL1 | T2D vs. no T2D aged≥70 | 6 | rs10946403 | 20717404 | G | A | 0.31 | 0.22 | 1.52 | 1.29 | 1.81 | 1.14E-06 |

SNPs: single nucleotide polymorphisms. CHR: chromosome. BP: Base Pair. A1: Alternate allele. A2: reference allele. P: P-value. OR: odd ratio. T2D: type 2 diabetes. L95 and U95: lower and upper limits of the 95% confidence interval for the OR estimate.

**Supplementary Table 4.** Replication of the association between the risk loci and T2D taking individuals without diabetes aged ≥65 or aged ≥70 as controls in the UK Biobank.

| **Study** | **Gene** | **CHR** | **SNP** | **BP** | **A1** | **OR** | **L95** | **U95** | **P** |
| --- | --- | --- | --- | --- | --- | --- | --- | --- | --- |
| T2D vs. no T2D aged≥65 | CDKAL1 | 6 | rs4712523 | 20657564 | G | 1.07 | 1.04 | 1.10 | 2.12E-07 |
| T2D vs. no T2D aged≥65 | CDKAL1 | 6 | rs7756992 | 20679709 | G | 1.10 | 1.07 | 1.13 | 9.28E-13 |
| T2D vs. no T2D aged≥70 | CDKAL1 | 6 | rs7756992 | 20679709 | G | 1.14 | 1.02 | 1.28 | 0.02 |

T2D: type 2 diabetes. SNPs: single nucleotide polymorphisms. CHR: chromosome. BP: Base Pair. A1: Alternate allele. P: P-value. OR: odd ratio. L95: lower bound of the 95% confidence interval for the odds ratio. U95: upper bound of the 95% confidence interval for the odds ratio.
